# Autistic traits and panic disorder severity: a retrospective six-month follow-up study

**DOI:** 10.3389/fpsyt.2026.1753142

**Published:** 2026-04-01

**Authors:** Yunus Akkeçili

**Affiliations:** Department of Psychiatry, Dinar State Hospital, Afyonkarahisar, Türkiye

**Keywords:** agoraphobia, autistic traits, broader autism phenotype, panic disorder, treatment outcome

## Abstract

Panic disorder (PD) exhibits marked clinical heterogeneity, and individual differences in autistic traits may contribute to variability in symptom severity and treatment course. This study examined whether autistic traits are associated with panic severity and agoraphobic avoidance during pharmacological treatment. In this retrospective observational study, 41 adults with DSM-5-diagnosed PD receiving guideline-based pharmacotherapy were followed over six months. Symptom trajectories were assessed using the Panic Disorder Severity Scale (PDSS) at baseline, one month, and six months, while autistic traits were measured using the Autism Spectrum Quotient (AQ) at the six-month visit. Linear mixed-effects models and repeated-measures ANCOVA examined associations between autistic traits and symptom burden while adjusting for age and sex. PDSS total and agoraphobia scores declined significantly over time (p <.001). Higher AQ total scores were associated with greater overall PD severity (p = .043) and more pronounced agoraphobic avoidance (p = .015) across assessments. Exploratory analyses indicated that attention switching and social skills were associated with overall severity, whereas reduced imagination was specifically linked to agoraphobic severity. Age was independently associated with agoraphobic severity but not with overall panic severity. No significant Time × AQ interactions were observed, indicating comparable symptom improvement across trait levels during the six-month treatment period. These findings suggest that elevated autistic traits are associated with persistently higher symptom burden during treatment without altering pharmacological response.

## 1 Introduction

Panic disorder (PD) is a recurrent anxiety condition characterized by sudden panic attacks, persistent anticipatory anxiety, and avoidance behaviors that frequently evolve into agoraphobia (1). Epidemiological studies estimate a lifetime prevalence of 3–5%, underscoring PD’s substantial public health burden (2). Despite the established efficacy of both pharmacological and cognitive-behavioral interventions, clinical outcomes remain heterogeneous. While factors such as baseline severity, comorbid depression, and disorder duration have been linked to less favorable prognoses, they do not fully account for the substantial clinical variance observed across patients (3). This unexplained heterogeneity suggests that additional individual characteristics, including autistic traits, may contribute to variability in clinical expression and symptom burden.

Autism spectrum disorder (ASD) is increasingly recognized within a dimensional framework rather than solely as a categorical diagnosis. The Broader Autism Phenotype (BAP) describes subthreshold autistic traits observed across the general population (4, 5). These traits, including differences in social reciprocity, cognitive rigidity, and atypical sensory sensitivity, are quantitatively distributed along a continuum. Genetic and phenotypic evidence suggests substantial overlap in liability for autistic traits across the general population and clinical-range presentations (6, 7). From a neurodiversity-informed perspective, such profiles may influence clinical presentations even without a formal ASD diagnosis, potentially shaping how co-occurring psychiatric conditions are experienced and reported.

Consistent with the dimensional model of neurodevelopment, recent evidence suggests that autistic traits are distributed across various psychiatric populations in a broad continuum, similar to the distribution observed in the general population, rather than being confined to clinical autism diagnoses (8). This dimensional perspective is particularly relevant for internalizing conditions, where elevated autistic traits, even at subthreshold levels, have been associated with greater symptom burden and impaired psychosocial functioning. Across psychiatric populations, autism-related trait dimensions have been linked to clinically meaningful variability, for example heightened avoidance in social anxiety (9), suicidality in mood disorders (10), and greater overall symptom severity and poorer psychosocial outcomes in obsessive–compulsive disorder (11, 12).

Empirical focus on autistic traits within PD has only recently gained momentum. Specifically, Dell’Osso et al. (13) identified elevated autism-spectrum features across panic, obsessive–compulsive, and social anxiety disorders, providing empirical support for a shared neurodevelopmental vulnerability underlying these conditions. Carpita et al. (14) further identified associations between specific autistic-trait domains and panic–agoraphobic symptom clusters. Nevertheless, the extent to which these traits relate to symptom trajectories during active treatment in longitudinal clinical samples remains to be elucidated.

Building on this emerging literature, the present study examined whether autistic traits are associated with panic disorder and agoraphobic severity in a clinical sample receiving routine pharmacological treatment. Using retrospectively extracted clinical assessments obtained at baseline, one month, and six months, we evaluated whether higher levels of autistic traits were associated with greater overall symptom burden and with symptom change over the six-month follow-up period after accounting for age and sex. We hypothesized that elevated autistic traits would be associated with greater panic and agoraphobia severity and would also be related to differences in the trajectory of symptom change during treatment.

## 2 Methods

### 2.1 Study design and participants

This study utilized a retrospective observational design conducted within a routine outpatient clinical setting. Participants were identified from the Psychiatry Outpatient Clinic of Dinar State Hospital, where individuals diagnosed with panic disorder (PD) were systematically assessed using the Panic Disorder Severity Scale (PDSS) at initial evaluation and subsequent follow-up visits as part of standard clinical care.

All participants received guideline-based pharmacotherapy (15, 16), primarily consisting of selective serotonin reuptake inhibitors (SSRIs) or serotonin–norepinephrine reuptake inhibitors (SNRIs). The most frequently prescribed regimens included escitalopram monotherapy, venlafaxine monotherapy, or venlafaxine augmented with aripiprazole. Additional strategies included SSRI or SNRI augmentation consistent with standard clinical protocols. A detailed distribution of pharmacological treatments maintained during the six-month follow-up period is provided in **Supplementary Table 1**.

At the six-month follow-up visit, individuals who provided written informed consent completed the Autism Spectrum Quotient (AQ) to assess autistic traits. PDSS scores from baseline, one-month, and six-month assessments were retrospectively extracted from medical records. Autistic traits are generally conceptualized as relatively stable characteristics. As the AQ was administered at the final assessment point, analyses were designed to evaluate associations between autistic traits and symptom levels across recorded follow-up assessments, rather than to test prospective prediction from baseline.

Eligible participants were adults aged 18–65 years with a DSM-5 diagnosis of PD who had completed at least six consecutive months of pharmacological treatment at the same center. Exclusion criteria included (1): receiving additional psychiatric medication or structured psychotherapy outside the standardized program during the preceding year (2); a current or lifetime diagnosis of psychotic disorder, bipolar disorder, or substance use disorder; and (3) significant cognitive impairment (e.g., dementia, intellectual disability) that could interfere with the valid completion of self-report measures. Furthermore, clinical evaluation records indicated that none of the participants had a documented DSM-5 diagnosis of Autism Spectrum Disorder, as the study focused on the distribution of subthreshold autistic traits.

**Figure 1:**
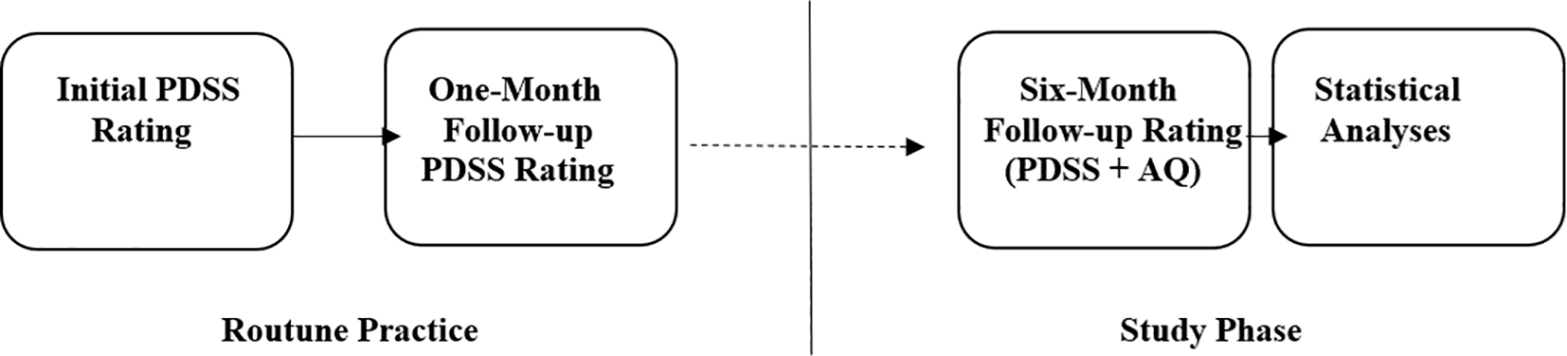
Flowchart of study design and assessment timeline. Participants with panic disorder (PD) were routinely evaluated using the Panic Disorder Severity Scale (PDSS) at baseline and one-month follow-up during standard clinical care. At the six-month visit, both PDSS and the Autism Spectrum Quotient (AQ) were administered as part of the study phase. Data from these three time points were analyzed using repeated-measures and linear mixed-effects models to examine associations between autistic traits and longitudinal symptom severity.

Ethical approval was granted by the Afyonkarahisar Health Sciences University Ethics Committee (Approval Date: December 13, 2024; Meeting No. 2024/11). A total of 41 participants met the eligibility criteria and were included in the final analyses (**Figure 1**).

### 2.2 Measures

#### 2.2.1 Autism spectrum quotient

The AQ is one of the most commonly used self-report measures for assessing autistic traits in adults. The adult version of the AQ consists of 50 items across five subscales: social skills, attention switching, attention to detail, communication, and imagination. It was developed to quantify autistic traits in neurotypical adults and identify individuals exhibiting traits consistent with autism spectrum characteristics. The Turkish version of the adult AQ was validated by Köse et al. (17).

In the Turkish validation study, the internal consistency of the AQ total score was reported as Cronbach’s α = 0.64, with subscale coefficients of 0.52 for Social Skills, 0.42 for Communication, 0.40 for Imagination, 0.52 for Attention to Detail, and 0.32 for Attention Switching (17).

#### 2.2.2 Panic disorder severity scale

The PDSS is a 7-item clinician-rated scale designed to assess the severity of panic disorder, including the frequency of panic attacks, anticipatory anxiety, agoraphobic avoidance, and functional impairment. Each item is rated on a 5-point Likert scale (0–4), yielding a maximum total score of 28. The Turkish adaptation and validation were conducted by Monkul et al. (18), confirming its reliability and validity for clinical use in PD populations. The Turkish validity and reliability study of the Panic Disorder Severity Scale reported high internal consistency, with Cronbach’s alpha coefficients ranging between 0.92 and 0.94. In the present study, the agoraphobia-related item of the PDSS was analyzed separately to assess agoraphobic avoidance severity; however, internal consistency could not be calculated for this subscore as it consists of a single item (18).

### 2.3 Statistical analysis

Statistical analyses were performed using IBM SPSS Statistics Version 25.0. Descriptive statistics were calculated for sociodemographic variables and clinical scores, while normality of distribution was evaluated using Shapiro–Wilk tests and visual inspection of Q–Q plots. Linear mixed-effects models (LMMs) were employed as the primary analytical framework to examine the longitudinal association between AQ total scores and PDSS trajectories. PDSS total and agoraphobia scores were specified as dependent variables, with time (baseline, one-month, and six-month) entered as a fixed effect. To account for potential demographic confounding at the primary level of analysis, age and sex were incorporated into the LMMs as fixed covariates. Models were estimated using restricted maximum likelihood (REML) with an unstructured covariance matrix to provide a flexible fit for longitudinal correlations.

Repeated-measures analyses of covariance (RM-ANCOVA) were conducted as secondary sensitivity and exploratory analyses. This dual-modeling approach served as a sensitivity check to evaluate the robustness of the primary findings while maintaining model parsimony for sub-dimension exploration. In the sensitivity models, AQ total scores were utilized as a covariate, while individual AQ subscales were examined in separate exploratory models to identify the specific autistic dimensions driving agoraphobic and panic symptom burden. Crucially, to ensure that these trait-specific associations were independent of demographic variance, age and sex were consistently incorporated as covariates in all RM-ANCOVA models. For these analyses, sphericity was assessed via Mauchly’s test, and Greenhouse–Geisser or Huynh–Feldt corrections were applied where appropriate. Effect sizes for between-subjects effects and interactions were reported as partial eta-squared (*η²_p_*), with values of.01,.06, and.14 representing small, medium, and large effects, respectively. All statistical tests were two-tailed, with a significance threshold of *p* < 0.05.

## 3 Results

### 3.1 Sociodemographic and descriptive statistics

A total of 41 individuals meeting DSM-5 criteria for panic disorder were included in the final analyses. The study population demonstrated a broad age range from 20 to 65 years, with a mean age of 40.6 years (SD = 10.8). Of the total sample, 41.5% of the participants were aged 45 years or older, representing a significant middle-aged to older adult subgroup within the clinical sample. Regarding gender distribution, 43.9% of the participants were male. The average duration of formal education was 9.7 years (SD = 3.6), and 56.1% of the sample reported a positive family history of psychiatric disorders. Sociodemographic characteristics are summarized in **Table 1**.

**Table 1:** Sociodemographic characteristics of participants.

Variable	Mean (SD)/n (%)	Range
Age (years)	40.61 (10.83)	20–65
Education (years)	9.73 (3.57)	4–16
Sex (Male/Female)	18 (43.9%)/23 (56.1%)	–
Family history of psychiatric disorder (Yes)	23 (56.1%)	–

This table summarizes the demographic and clinical characteristics of the study sample (n = 41). Values are presented as mean ± standard deviation (SD) for continuous variables and as number (percentage) for categorical variables.

The mean Autism Spectrum Quotient (AQ) total score was 21.9 (SD = 5.4), reflecting the distribution of autistic traits within the clinical sample. The specific subscale distributions were characterized by scores in social skills (M = 4.1, SD = 1.9), attention switching (M = 4.8, SD = 2.0), attention to detail (M = 5.1, SD = 1.9), communication (M = 2.9, SD = 1.6), and imagination (M = 4.9, SD = 1.2), as presented in **Table 2**.

**Table 2:** Descriptive statistics of AQ total and subscales.

AQ subscale	Mean (SD)	Range
Social Skills	4.12 (1.87)	1–8
Attention Switching	4.76 (2.05)	1–9
Attention to Detail	5.05 (1.92)	1–9
Communication	2.90 (1.56)	0–8
Imagination	4.93 (1.23)	2–7
AQ Total	21.88 (5.39)	11–35

This table presents the mean, standard deviation, and range for total and subscale scores of the Autism Spectrum Quotient (AQ). Higher scores indicate greater expression of autistic traits within each respective domain.

**Figure 2:**
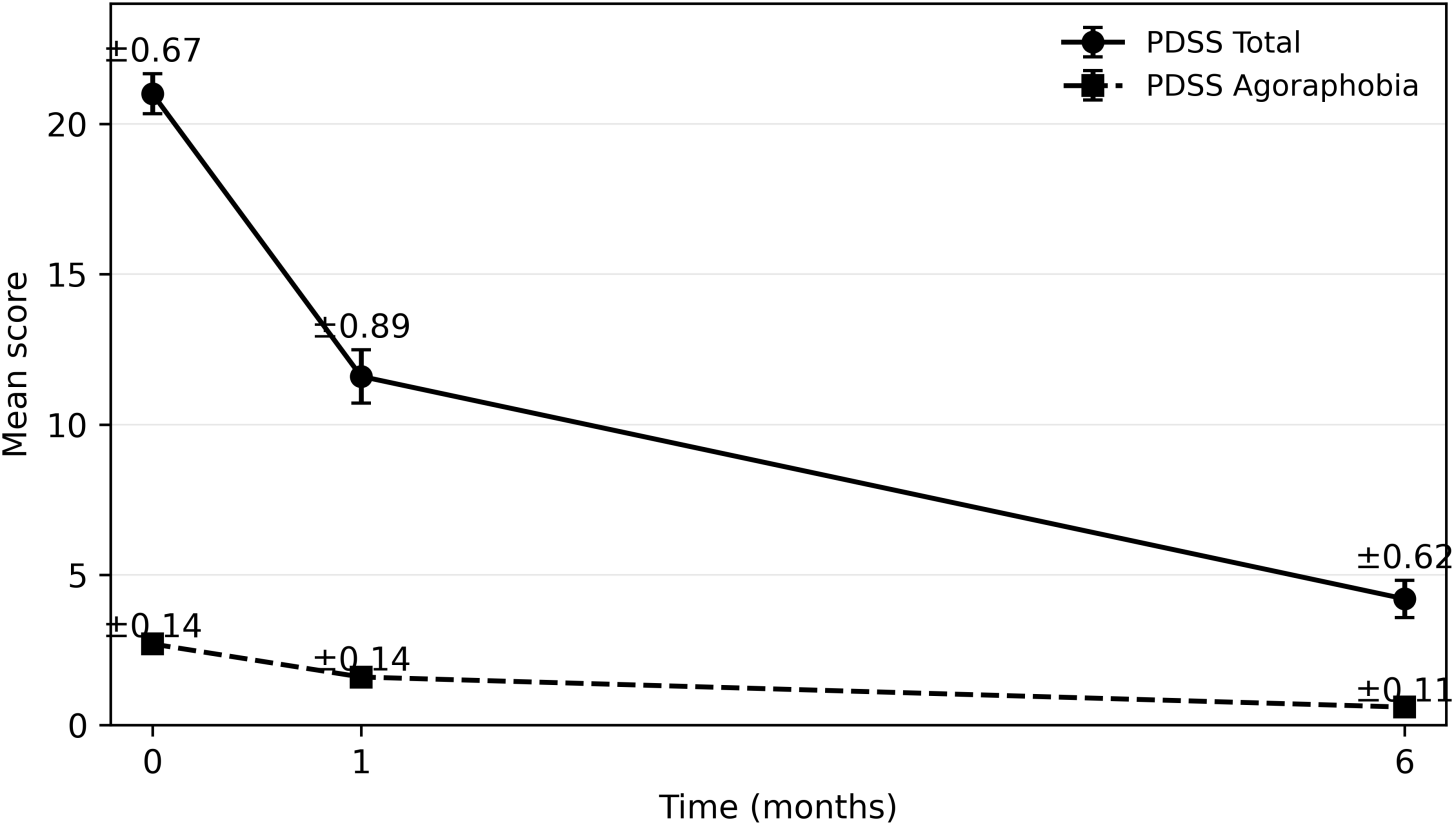
Longitudinal change in PDSS total and agoraphobia scores. Mean PDSS total and agoraphobia scores are plotted across baseline, one-month, and six-month assessments. Error bars represent standard errors of the mean (SEM). Both indices show a significant decrease over time (p < 0.001 for main effect of time), indicating progressive symptom improvement under routine pharmacological treatment.

Clinical severity, measured by the Panic Disorder Severity Scale (PDSS), showed a marked reduction throughout the treatment period. The mean PDSS total score decreased from baseline (M = 21.0, SD = 4.3) to the one-month (M = 11.6, SD = 5.7) and six-month (M = 4.2, SD = 4.0) assessments. Similarly, agoraphobic severity scores exhibited a consistent decline from baseline (M = 2.7, SD = 0.9) to one month (M = 1.5, SD = 1.0) and reached their lowest levels at the six-month follow-up (M = 0.6, SD = 0.7). Descriptive values for these clinical trajectories are displayed in **Figure 2**.

### 3.2 Linear mixed-effects models (primary analyses)

Linear mixed-effects models were conducted to examine the longitudinal association between autistic traits and panic severity while adjusting for age and sex. Regarding PDSS total severity, a significant main effect of time was observed, *F (2*, 39) = 21.84, *p* <.001. The AQ total score demonstrated a significant association with overall PDSS severity, *F*(1, 36.61) = 4.41, *p* = .043, where higher AQ total scores were associated with higher PDSS severity across assessments. The time × AQ interaction was not significant (*p* = .566), and neither age (*p* = .300) nor sex (*p* = .671) emerged as significant predictors in this model.

For PDSS agoraphobia subscores, a significant main effect of time was observed, *F*(2, 41) = 8.05, *p* = .001. The AQ total score was significantly associated with agoraphobic severity, *F*(1, 40.38) = 6.42, *p* = .015. In this model, age was also found to be significantly associated with agoraphobic severity, *F*(1, 40.99) = 4.84, *p* = .034, whereas sex was not a significant factor (*p* = .457). The time × AQ interaction for agoraphobia was non-significant, *F*(2, 41) = 0.01, *p* = .990.

Full LMM results and model fit indices (AIC, BIC) are presented in **Supplementary Table 2**.

### 3.3 Repeated-measures ANCOVA (sensitivity analyses)

Repeated-measures ANCOVA (RM-ANCOVA) were performed as sensitivity analyses to examine the stability of the primary findings while adjusting for age and sex. For PDSS total severity, the model revealed a significant main effect of time, *F*(1.65, 61.23) = 9.56, *p* = .001, *η²_p_* = .205. After controlling for age and sex, the AQ total score demonstrated a significant independent association with overall symptom burden across all time points, *F*(1, 37) = 8.37, *p* = .006, *η²_p_* = .184. İn this model, neither age nor sex emerged as significant covariates regarding total symptom severity (all *p* >.05).The Time × AQ interaction effect was not significant, *F*(1.65, 61.23) = 0.90, *p* = .397, *η²_p_* = .024.

For agoraphobia subscores, the RM-ANCOVA identified a significant between-subjects effect for the AQ total score, *F*(1, 37) = 7.78, *p* = .008, *η²_p_* = .174. In this adjusted model, age also emerged as a significant covariate, *F*(1, 37) = 6.17, *p* = .018, *η²_p_* = .143, whereas sex did not show a significant association with agoraphobic severity (*p* = .168). While the main effect of time for agoraphobia trended toward significance in the within-subjects test, *F*(2, 74) = 2.59, *p* = .082, *η²_p_* = .065, multivariate testing and *post-hoc* pairwise comparisons revealed a highly significant reduction in agoraphobic symptoms over time. Specifically, Bonferroni-adjusted pairwise comparisons demonstrated significant symptomatic improvement between all assessment points (Baseline to Month 1, Month 1 to Month 6, and Baseline to Month 6; all *p* <.001). The Time × AQ interaction for agoraphobia was non-significant, *F*(2, 74) = 0.04, *p* = .965, *η²_p_* = .001.

### 3.4 Exploratory subscale analyses

Exploratory RM-ANCOVA models, adjusted for age and sex, were conducted for both PDSS total and agoraphobia subscale scores to pinpoint specific autistic dimensions driving the clinical associations.

For overall PDSS severity, attention switching demonstrated the largest effect size *F*(1, 37) = 11.86, *p* = .001, *η²_p_* = .243, followed by social skills *F*(1, 37) = 4.64, *p* = .038, *η²_p_* = .111. In contrast, imagination, communication, and attention to detail did not show significant independent associations with total symptom burden (all *p* >.05). Notably, age was identified as a significant covariate primarily in the attention switching model (*p* = .028), while it remained non-significant in the other models for PDSS total.

For agoraphobia severity, the associations were more extensive, with social skills *F*(1, 37) = 9.12, *p* = .005, *η²_p_* = .198, attention switching *F*(1, 37) = 8.06, *p* = .007, *η²_p_* = .179, and imagination *F*(1, 37) = 4.96, *p* = .032, *η²_p_* = .118 emerging as significant independent predictors. Notably, age remained a robust independent covariate across every agoraphobia model tested, consistently demonstrating a significant association with agoraphobic severity regardless of the specific AQ dimension included (e.g., in the social skills model: *F*(1, 37) = 6.50, *p* = .015, *η²_p_* = .149; and even in the non-significant communication model: *F*(1, 37) = 5.31, *p* = .027, *η²_p_* = .126). In contrast, the AQ dimensions of communication and attention to detail did not reach statistical significance (*p* >.05) in their respective models.

Across all adjusted models, no significant Time × Subscale interaction effects were identified (all *p* >.05), and sex did not emerge as a significant factor in any analysis (*p* >.05). Full results for all subscale models are provided in **Supplementary Table 3**.

## 4 Discussion

### 4.1 Autistic traits and panic disorder severity in a dimensional framework

The present study examined the association between autistic traits and symptom severity in panic disorder (PD) across a six-month period of pharmacological treatment. Two primary findings were observed. First, higher levels of autistic traits were consistently associated with greater overall PD severity and more pronounced agoraphobic avoidance across all assessment points, independent of age and sex. Second, autistic traits were not associated with differences in the rate of symptom improvement under pharmacotherapy, as indicated by the absence of significant Time × Trait interaction effects.

These findings are consistent with recent research suggesting overlap between panic-spectrum psychopathology and autism-related trait dimensions (13, 14). The present results extend this emerging literature by indicating that although pharmacological treatment was associated with substantial symptom reduction over time, individuals with higher autistic-trait levels maintained higher overall symptom severity across follow-up assessments. This pattern suggests that autistic traits are associated with baseline and concurrent symptom burden rather than with diminished responsiveness to medication.

With regard to demographic factors, age was significantly associated with agoraphobic severity but not with overall panic severity. This differential pattern may indicate that avoidance-related phenomena are more sensitive to age-related factors than core panic symptoms. One possible interpretation is that agoraphobic avoidance behaviors may consolidate over time; however, the present design does not allow for direct examination of developmental or interaction effects. Prospective longitudinal studies beginning earlier in the course of disorder are needed to clarify how age-related processes intersect with autistic trait dimensions in shaping agoraphobic symptom patterns.

### 4.2 Mechanisms linking autistic traits to panic–agoraphobic severity

The dimension-specific associations observed in our exploratory analyses can be interpreted within the context of prior dimensional studies examining the overlap between autistic traits and panic–agoraphobic symptomatology. Previous work has consistently demonstrated positive correlations between autism-spectrum domains and panic-agoraphobic symptom clusters in clinical samples (14). Our findings appear consistent with this emerging literature.

In particular, attention switching and social skills showed the strongest associations with both overall panic severity and agoraphobic subscores. Although the present study did not directly test cognitive mechanisms, reduced attentional flexibility has been described as a core autistic-trait dimension and has been linked in prior literature to difficulties in adapting to changing environmental demands. Within panic disorder, such reduced flexibility may be relevant to the persistence of threat-focused attention or difficulty integrating safety information, processes central to contemporary cognitive models of panic.

Similarly, the association between imagination scores and agoraphobic severity aligns with prior dimensional findings suggesting that specific autistic-trait domains may relate differentially to panic–agoraphobic symptom clusters. Rather than implying a direct causal pathway, this pattern may reflect that certain trait dimensions are more closely linked to avoidance-related processes than to panic frequency per se.

In addition, emotion regulation differences and heightened interoceptive sensitivity, which are frequently described in both autism and panic models, may contribute to amplified salience of bodily sensations and anticipatory anxiety, potentially reinforcing catastrophic interpretations and avoidance behaviors. These mechanisms were not directly tested in the present study, and therefore remain conceptual rather than empirical explanations of the observed associations.

Previous literature has also highlighted the role of sensory hyper- or hyporeactivity, intolerance of uncertainty, and cognitive inflexibility in the association between autistic traits and panic–agoraphobic symptoms (14). In this framework, agoraphobic avoidance may be understood as part of a broader pattern of heightened sensitivity to environmental unpredictability and rigid coping strategies within a dimensional neurodevelopmental vulnerability model. Our findings are consistent with this perspective, indicating that specific autistic dimensions may relate more strongly to agoraphobic manifestations than to global panic severity.

From a broader autism perspective, these results align with evidence describing elevated anxiety, sensory sensitivity, and cognitive rigidity among autistic individuals. Although the present sample did not include participants with a formal autism spectrum disorder diagnosis, the dimensional distribution of autistic traits suggests potential continuity between subthreshold trait levels and clinically diagnosed autism. Examination of panic symptomatology in formally diagnosed autistic populations will be important for determining whether similar severity patterns are observed at higher trait levels.

Taken together, these results support the view that autistic traits may not merely co-occur with panic disorder, but may shape the phenomenological expression of panic–agoraphobic symptoms in a dimension-specific manner. However, given the correlational and retrospective nature of the present design, these interpretations should be considered hypothesis-generating and warrant further prospective investigation.

### 4.3 Symptom burden versus treatment trajectory

An additional clinically relevant observation concerns the relationship between autistic traits and treatment trajectory. Although autistic traits were associated with consistently higher levels of panic and agoraphobic severity across assessment points, they were not associated with differential rates of symptom change over time. Thus, autistic trait levels did not appear to influence the trajectory of symptom improvement during treatment. The absence of significant Time × Trait interaction effects suggests that the trajectory of clinical improvement under pharmacological treatment was comparable across varying levels of autistic traits.

This pattern indicates that while autistic traits are linked to overall symptom burden, they do not appear to modify pharmacological response over the six-month treatment period. Individuals with higher trait levels showed parallel reductions in symptoms despite starting from higher baseline severity.

These findings differ from some psychotherapy-based studies reporting attenuated cognitive-behavioral therapy outcomes among individuals with elevated autistic traits (19, 20). One possible explanation is that pharmacological interventions primarily target neurobiological mechanisms underlying autonomic arousal and anxiety sensitivity, processes that may be less directly dependent on cognitive flexibility or social-cognitive processing. However, this interpretation should be considered cautiously, as the present study was not designed to directly compare treatment modalities.

If replicated in samples including formally diagnosed autistic individuals, these findings may have broader implications for understanding how different intervention modalities interact with neurodevelopmental traits across psychiatric conditions.

Clinically, the results suggest that autistic traits may contribute to a persistently higher symptom burden without necessarily constituting a barrier to pharmacological efficacy. Patients with elevated autistic traits demonstrated meaningful symptom improvement over time, even if residual symptom levels remained comparatively higher.

### 4.4 Interpretation of AQ assessment and self-report considerations

The timing of AQ administration at the six-month follow-up represents an important methodological consideration. Although autistic traits are generally conceptualized as relatively stable characteristics (4), the assessment occurred after substantial symptom improvement had already taken place. As a result, AQ scores cannot be interpreted as baseline predictors of symptom trajectory.

It is possible that changes in symptom severity, treatment experience, increased insight, or shifts in self-perception during the treatment period influenced self-reported trait scores. At the same time, the convergence of findings across two independent analytical frameworks (LMM and RM-ANCOVA), both adjusted for age and sex, supports the internal consistency of the observed trait–severity associations within this sample. Nevertheless, the retrospective structure of the design limits firm conclusions regarding temporal directionality.

Prospective studies incorporating trait assessment at treatment initiation, alongside repeated longitudinal measurement, will be necessary to clarify the stability of these associations and their predictive relevance during early treatment phases.

### 4.5 Strengths, limitations, and clinical implications

This study has several methodological strengths. The longitudinal assessment of panic severity across three time points allowed for examination of symptom trajectories rather than single cross-sectional comparisons. The use of linear mixed-effects modeling, complemented by sensitivity analyses with RM-ANCOVA, provided a robust analytical framework while consistently adjusting for age and sex. Furthermore, the investigation of autistic traits within a routine outpatient setting enhances ecological validity and reflects real-world psychiatric practice.

Several limitations should be acknowledged. First, the retrospective design precludes causal inference and limits interpretation of autistic traits as prospective predictors of treatment outcome. Second, the relatively modest sample size reduces statistical power for detecting subtle interaction effects. Third, inclusion of treatment completers only may introduce survivorship bias, as individuals with more severe symptom profiles or higher neurodevelopmental rigidity who discontinued treatment were not captured. The study was designed to examine the dimensional distribution of autistic traits within a clinically diagnosed panic disorder sample rather than to investigate autism as a categorical condition. Accordingly, the findings pertain to subthreshold and trait-level variability within PD. Finally, although the sample was derived from routine clinical practice, replication in larger and more diverse cohorts would strengthen the generalizability of the observed associations.

From a clinical perspective, the findings suggest that elevated autistic traits are associated with a persistently higher symptom burden across the treatment period, without evidence of differential response slopes under pharmacotherapy. Recognition of dimensional neurodevelopmental trait profiles may contribute to more individualized clinical formulation, particularly in the context of residual agoraphobic symptoms rather than prospective prediction. Future research should determine whether adjunctive, trait-informed interventions can further optimize long-term functional outcomes.

## Data Availability

The raw data supporting the conclusions of this article will be made available by the authors, without undue reservation.
