# Supplementary material for: Autistic traits and panic disorder severity: a retrospective six-month follow-up study
Source: Front Psychiatry. 2026 Apr 1;17:1753142. doi: 10.3389/fpsyt.2026.1753142 (PMC13079360; doi:10.3389/fpsyt.2026.1753142)
Supplement: Supplementary file 1 [file SupplementaryFile1.docx]

**Supplementary Material**

This supplementary material provides additional methodological and statistical details supporting the main findings of the study entitled " Autistic Traits and Panic Disorder Severity: A Retrospective Six-Month Follow-Up Study." Within this document, Supplementary Table S1 details the pharmacological treatment distribution among participants at the six-month follow-up assessment point. Supplementary Table S2 presents the comprehensive statistical results and model fit indices for the Linear Mixed-Effects Models assessing longitudinal changes in PDSS scores while controlling for age and sex as fixed-effects covariates. Finally, Supplementary Table S3 provides the Repeated-Measures ANCOVA results examining the association between individual Autism Spectrum Quotient dimensions and both total PDSS and agoraphobic severity while consistently controlling for age and sex as independent covariates. All statistical analyses were conducted using IBM SPSS Statistics Version 25.0.

**Supplementary Table S1. Pharmacological Treatment Distribution among PD Participants**

| **Treatment Type** | **n** | **%** | **Description** |
| --- | --- | --- | --- |
| Escitalopram monotherapy | 14 | 34.1 | SSRI monotherapy |
| Venlafaxine monotherapy | 8 | 19.5 | SNRI + antipsychotic augmentation |
| Venlafaxine + Aripiprazole | 8 | 19.5 | SNRI ± antipsychotic augmentation |
| SSRI + antipsychotic | 5 | 12.2 | SNRI ± antipsychotic augmentation |
| Paroxetine or Sertraline monotherapy | 3 | 7.3 | SSRI monotherapy |
| Other | 3 | 7.3 | Mixed regimens within standard clinical protocols (e.g., SSRI/SNRI + antipsychotic or antidepressant) |

**Note:** Data reflect the primary pharmacological regimen maintained for most of the six-month follow-up period. All treatments were administered within routine clinical practice following guideline-based pharmacological recommendations for panic disorder (*n = 41*).

**Supplementary Table S2. Linear Mixed-Effects Model (LMM) Results for PDSS Severity and Agoraphobia**

| **Model** | **Fixed Effect** | ***F (df)*** | ***p*** | ***AIC*** | ***BIC*** |
| --- | --- | --- | --- | --- | --- |
| **PDSS Total Severity** | **Time** | **21.837 (2, 39.00)** | **<.001** | 675.423 | 694.638 |
|  | **AQ Total** | **4.413 (1, 36.61)** | **.043** |  |  |
|  | Age | 1.107 (1, 37.00) | .300 |  |  |
|  | Sex | 0.183 (1, 37.00) | .671 |  |  |
|  | Time × AQ | 0.577 (2, 39.00) | .566 |  |  |
| **PDSS Agoraphobia** | **Time** | **8.052 (2, 41.00)** | **.001** | 266.895 | 309.078 |
|  | **AQ Total** | **6.421 (1, 40.38)** | **.015** |  |  |
|  | **Age** | **4.837 (1, 41.00)** | **.034** |  |  |
|  | Sex | 0.564 (1, 41.00) | .457 |  |  |
|  | Time × AQ | 0.010 (2, 41.00) | .990 |  |  |

**Note:** Linear mixed-effects models (LMM) were estimated using restricted maximum likelihood (REML) with a random intercept for each participant. Denominator degrees of freedom were estimated using the Satterthwaite approximation. Age, sex, and AQ total scores were entered as fixed-effects covariates. Consistent with the primary findings, Time and AQ Total scores were significant independent predictors for both models, while age was a specific significant covariate for agoraphobic severity. Model fit is indicated by Akaike’s Information Criterion (AIC) and Schwarz’s Bayesian Criterion (BIC).

**Supplementary Table S3. RM-ANCOVA Results for PDSS Severity with AQ Subscales and Age as Covariates**

| **PDSS Domain (Within-Subject)** | **AQ Subscales (Between-Subject)** | ***F*(1,37)** | ***p*** | ***η²ₚ*** | **Age *F*(*p*)** |
| --- | --- | --- | --- | --- | --- |
| **PDSS Total Severity** | **Attention Switching** | **11.855** | **.001** | **.243** | **5.229 (.028)** |
|  | **Social Skills** | **4.638** | **.038** | **.111** | 3.566 (.067) |
|  | Communication | 3.234 | .080 | .080 | 3.260 (.079) |
|  | Imagination | 1.298 | .262 | .034 | 2.812 (.102) |
|  | Attention to Detail | 0.355 | .555 | .009 | 3.147 (.084) |
| **PDSS Agoraphobia** | **Social Skills** | **9.117** | **.005** | **.198** | **6.499 (.015)** |
|  | **Attention Switching** | **8.065** | **.007** | **.179** | **7.398 (.010)** |
|  | **Imagination** | **4.959** | **.032** | **.118** | **4.724 (.036)** |
|  | Communication | 1.917 | .174 | .049 | 5.315 (.027) |
|  | Attention to Detail | 1.359 | .251 | .035 | 5.259 (.028) |

**Note:** Each row represents a separate RM-ANCOVA model including age and sex as covariates. *F* and *p* values for AQ subscales and **Age *F*(*p*)** indicate their unique effects on symptomatic severity. Partial *η²ₚ* > .14 denotes a large effect size. Degrees of freedom (1, 37) apply to all reported between-subjects tests (AQ subscales and age).
